# Modulation of voltage-gated Ca_V_2.2 Ca^2+^ channels by newly identified interaction partners

**DOI:** 10.1080/19336950.2020.1831328

**Published:** 2020-10-15

**Authors:** Lubica Lacinova, Robert Theodor Mallmann, Bohumila Jurkovičová-Tarabová, Norbert Klugbauer

**Affiliations:** a Center of Bioscience, - Institute for Molecular Physiology and Genetics, Bratislava, Slovakia; b Faculty of Natural Sciences, University of Ss. Cyril and Methodius, Trnava, Slovakia; c Institut für Experimentelle und Klinische Pharmakologie und Toxikologie, Fakultät für Medizin, Albert-Ludwigs-Universität Freiburg, Freiburg, Germany; d Center for Basics in NeuroModulation (Neuromodul Basics), Albert-Ludwigs-Universität Freiburg, Freiburg, Germany

**Keywords:** calcium channel, Cav2.2, G protein, Grina/TMBIM3, channel modulation

## Abstract

Voltage-gated Ca^2+^ channels are typically integrated in a complex network of protein-protein-interactions, also referred to as Ca^2+^ channel nanodomains. Amongst the neuronal Ca_V_2 channel family, Ca_V_2.2 is of particular importance due to its general role for signal transmission from the periphery to the central nervous system, but also due to its significance for pain perception. Thus, Ca_V_2.2 is an ideal target candidate to search for pharmacological inhibitors but also for novel modulatory interactors. In this review we summarize the last years findings of our intense screenings and characterization of the six Ca_V_2.2 interaction partners, tetraspanin-13 (TSPAN-13), reticulon 1 (RTN1), member 1 of solute carrier family 38 (SLC38), prostaglandin D2 synthase (PTGDS), transmembrane protein 223 (TMEM223), and transmembrane BAX inhibitor motif 3 (Grina/TMBIM3) containing protein. Each protein shows a unique way of channel modulation as shown by extensive electrophysiological studies. Amongst the newly identified interactors, Grina/TMBIM3 is most striking due to its modulatory effect which is rather comparable to G-protein regulation.

## Introduction

Ca_V_2.2, or, according to an earlier nomenclature, N-type calcium channels, belong to the high-voltage-activated Ca^2+^ channels [1]. According to their physiological and pharmacological properties, they were first characterized in the 1980s by the Tsien group [2]. The cDNA encoding the principal α_1_ subunit of this channel was first cloned in 1990 [3]. Transcripts of the CACNA1B gene were found almost exclusively in central and peripheral neurons [2] predominantly in dendritic shafts and presynaptic terminals [4]. Particularly high expression levels were found in the superficial layer of the dorsal horn, i.e., in the putative nociceptive area of the spinal cord [5]. Their localization predetermines the role of Ca_V_2.2 channels in transmission of signals from peripheral neurons to the central nervous system. Ca^2+^ entry through presynaptic Ca_V_2.2 channels initiates a complex cascade of events leading to a fusion of exocytotic vesicles with the presynaptic membrane and release of neurotransmitters into the synaptic cleft. Within the last decades, an impressive high number of proteins located at transmitter release sites in presynaptic terminals were identified, that directly interact with Ca_V_2.2 channels. These include α-catenin, N-ethylmaleimide-sensitive factor, Munc18, vesicle-associated membrane protein, α-Spectrin, and others [6]. Modulation of Ca^2+^ currents through this channel, therefore, modulates also signal transmission through the respective synapse.

Perception transmitted by these channels is not restricted to various pain signals but also includes sensations like touch, itch, and body muscle tension [7]. Experiments with constitutive knockouts of the CACNA1B gene demonstrated a predominant role of Ca_V_2.2 channels in nociception but a minor contribution to physiological processes like sympathetic control of the cardiovascular system, which makes them an attractive target for pharmacological pain treatment by suppression of Ca^2+^ entry through Ca_V_2.2 channels. Therefore, it is highly desirable to elucidate modulatory mechanisms of Ca_V_2.2 channels by various ligands and/or pathways to distinguish between gain of function and loss of function features.

**Figure 1.**
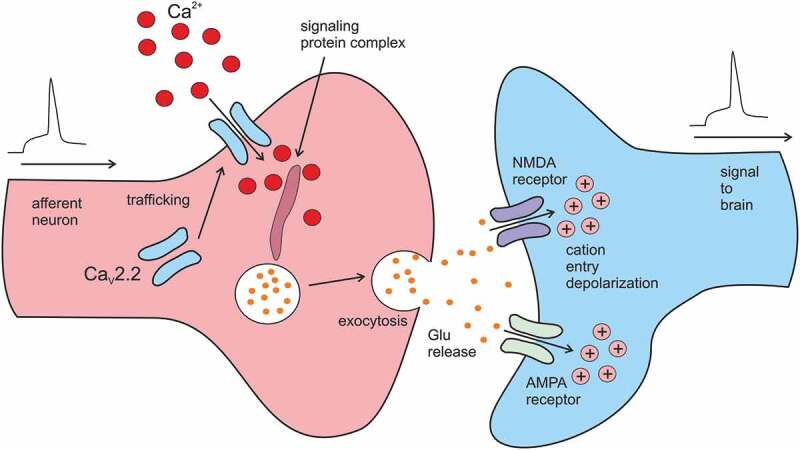
Schematic depiction of the role of presynaptic Ca_V_2.2 in signal transmission in a nociceptive synapse. Modified from [22] with permission.

Proteins affecting Ca^2+^ entry through Ca_V_2.2 channels may either alter gating and/or conductance of the channel protein or may disrupt trafficking of the channel subunits into the cell membrane. Recently, MAP6 was shown to interact directly with the C-terminus of Ca_V_2.2 channels and to participate in channel trafficking as the amount of channels in neurons from MAPK6 KO mice was decreased by 25% [8]. The Ca_V_2.2 C-terminus also directly interacts with the PDZ domain of RIM proteins and this interaction is essential for tethering Ca_V_2.2 channels at presynaptic terminals where Ca^2+^ influx through these channels triggers neurotransmitter release [9]. Both mechanisms, i.e., alteration of channel trafficking and channel gating, may be exploited to manipulate Ca_V_2.2 mediated Ca^2+^ signaling (Figure 1).

### Modulation of Ca_V_2.2 channels

Organic toxins, e.g., conotoxins, isolated from marine snails or Cd1a toxin from the African spider *Ceratogyrus darlingi*, mostly block the channel pore [10] and modulate voltage dependence and/or current kinetics to a minor extent (Table 1). Similar mechanisms were reported for conotoxin FVIA from *Conus fulmen* [11] and for ω-conotoxins CVIE and CVIF from *Conus catus* [12]. The 4-amino-piperidine derivative ZC88 inhibits Ca_V_2.2 channels in a state-dependent manner, preferentially by blocking the inactivated state of the channel [13].

**Table 1.** Interaction of peptide toxins and proteins with the Ca_V_2.2 channel tested by means of electrophysiology. Meaning of symbols: ND – not determined; ↔ non-significant effect; ↑ – statistically significant enhancement (acceleration of activation/inactivation kinetics, increase in extent of cumulative inactivation, increased current facilitation by prepulse); ↓ – statistically significant suppression (lower current amplitude, slowed kinetics of activation, smaller extent of cumulative inactivation, reduced current facilitation by prepulse). Modulations resulting in “loss-of-function” are highlighted in red, “gain-of-function” modulations are marked in green.

Protein	Current amplitude	Voltage dependence of activation/inactivation	Kinetics of current activation/inactivation	Cumulative inactivation	Prepulse facilitation
^a^ω-conotoxin MVIIA	**↓**		ND/**↑**		
^b^ω-conotoxin GVIA	**↓**	**←**/ND	**↔/**ND		
^c^Cd1a	**↓**	**↔/←**	**↔/↔**		
^d^conotoxins FVIA, CVIE, and CVIF	**↓**				
^e^ZC88	**↓**	**↔/←**	**↔/↔**		
^f^Protein kinase C	**↑**		ND/**↑**		**↔**
^g^GPCR Muscarinic receptor	**↓**	**↔**/ND	**↓/↓**		**↑**
^h^GPCR GTP-γ-S	**↓**	**→**/ND	**↓/↓**	**↓**	**↑**
^i^GPCR opioid receptors	**↓**	**→**/ND	**↓**/ND	**↓**	**↑**
^j^GPCR µ-opioid receptor	**↓**	**→**/ND	**↓**/ND		**↑**

a – [69]; b – [70]; c – [71]; d – [11,12]; e – [13]; f – [72]; g – [61]; h – [30,31]; i – [62]; j – [63]

Phosphorylation of the α_1_ subunit by protein kinase C (PKC) results in an enhancement of current amplitude and an acceleration of current inactivation (Table 1). The Ca_V_2.2 amino acid residues T422, S1757, S2108, and S2132 were identified as PKC phosphorylation sites [14,15]. Channel phosphorylation by PKC is the only known endogenous modulation resulting in an enhanced calcium entry (Table 1). It was suggested that phosphorylation by PKC relieves inhibition of the channel by G-proteins [16–18]. Phosphorylation of Thr422, located in the intracellular loop connecting domains I and II of the α_1_ subunit, underlies this effect [19].

Ca^2+^ currents through Ca_V_2.2 channels may be negatively modulated by agonists of G-protein-coupled receptors (GPCRs). GPCRs represent a many-faceted group of more than 1000 receptors activated by a wide range of substances, including light, hormones, amines, neurotransmitters, and lipids. For modulation of Ca_V_2.2 channels, synaptically located receptors like opioid or muscarinic receptors are of importance. Upon activation by agonist binding to GPCRs, the heterotrimeric G-protein splits into a G_α_ monomer and a Gβγ dimer. Gβγ dimers directly bind to the α_1_ subunit of Ca_V_2.2 and negatively modulate the current through these channels. For instance, this basic mechanism is causal for the antinociceptive effect of opioids.

### Mechanism of inhibition of Ca_V_2.2 channels by G-proteins

Modulation of the N-type Ca^2+^ channels by G-proteins is most extensively studied and was recently reviewed by several authors [20–23]. To describe the alteration of channel gating upon interaction with G-proteins, a model of “willing” and “reluctant” gating states was introduced [24,25]. A non-modified “willing” gating mode is characterized by fast channel opening. In the “reluctant” gating state, channel opening is slower and requires higher depolarization, i.e., current–voltage relation is shifted toward more positive membrane potentials [24,25]. There is a broad agreement that the “reluctant” channel state is due to a direct binding of Gβγ to the Ca_V_2.2 α_1_ subunit [23,26,27]. A hallmark of G-protein mediated inhibition of N-type Ca^2+^ channels is its relief by brief depolarizing pulses of high amplitude, called prepulse facilitation [24,28]. Such depolarization ensures a rapid conformational transition of all channels into an open state, which destabilizes Gβγ–binding to the channel [28]. However, large depolarizations used in prepulse facilitation paradigms are non-physiological. Under physiological conditions, Ca_V_2.2 channels are activated by action potentials or by trains of action potentials. Such experimental protocols were able to relieve G-protein-related current inhibition to a certain extent [29–32].

**Figure 2.**
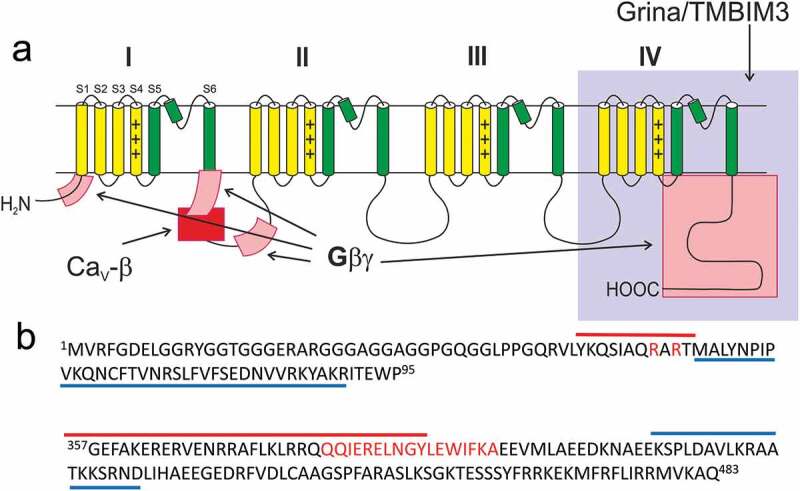
Schematic representation of the Ca_V_2.2 α1 subunit (a) and parts of amino acid sequence of rat Ca_V_2.2 α1 subunit (UniProtKB – Q02294) (b). Three loci (pink blocks in A) were identified as putative interaction sites with Gβγ subunits. Site on N-terminus (amino acids 1–95) includes amino acids 45–55 (red line in B) [36] with specific role of amino acids 52 and 54 [37] and amino acids 56–90 (blue line in B) [35]. Two interaction sites were identified in the loop connecting domains I and II. First part overlaps with the α1-interaction site (red block in A) consisting of amino acids 379–396 (red letters in B) [33] and involves amino acids 353–389 (red line in B) [18,34]. Depicted is amino acid sequence of the I–II loop. The entire interaction site includes also amino acids ^353^GVLS^356^ from the IS6 segment. Second is located further downstream and includes amino acids 410–428 (blue line in B) [18]. Role of C-terminus is less clear and putative binding region was not unambiguously identified. Domain IV interacts with Grina/TMBIM3 (gray block in A) but is not narrowed down in more detail.

Three regions were identified in the α_1_ subunit of Ca_V_2.2 as possible sites for interactions with Gβγ subunits of G-proteins (Figure 2): an intracellular loop connecting domains I and II and parts of the N and C-terminus [20,21]. Further, two distinct interaction sites were identified in the I–II loop. The first one partially overlaps with the well-established β-subunit interaction site (AID) [33], while the second one is located downstream of AID and was designated as G protein interaction domain (GID) [18,23,34]. The N-terminus not only binds Gβγ subunits, but also directly interacts with the I–II loop in the presence of Gβγ subunits forming the “reluctant” channel configuration [35]. The second half of the N-terminus was shown to be more important for an interaction with Gβγ subunits [36,37]. The role of the C-terminus in G-protein-mediated inhibition is less prominent [19,38,39]. It has been suggested that all three putative interaction sites together form a G-protein binding pocket (GPBP) [40]. This model is based on kinetics studies of Gβγ interaction with the Ca_V_2.2 channel, which support a 1:1 stoichiometry [27].

### Identification of Ca_V_2.2 interaction partners

The interactome of voltage-gated Ca^2+^ channels has been investigated by a variety of comprehensive biochemical and molecular biological techniques. Affinity purification in combination with quantitative mass spectrometry allowed a detailed characterization of Ca_V_2 Ca^2+^ channel nanodomains [41,42]. More than 200 putative interaction partners have been identified by Müller and colleagues by using a proteomic strategy based on multiepitope affinity purifications with high-resolution quantitative MS. However, the experimental approaches selected might favor the identification of certain protein–protein interactions and depend on the biochemical properties of the interactors. Yeast two-hybrid (YTH) assays have also been applied as independent and unbiased methods to screen for novel interaction partners. For instance, studies using the classical YTH system identified active zone RIM proteins as Ca^2+^ channel tethering adapter molecules [9]. However, these YTH systems were primarily developed to screen for interactions between soluble proteins and not for those between hydrophobic transmembrane domains such as Ca^2+^ channels. Therefore, we took advantage of a modified YTH system that allows detection of interactions between membrane proteins [43]. This assay relies on the use of a split-ubiquitin. Only the interaction of two proteins, which are fused either to the N- or C-terminal, half of split ubiquitin reconstitutes ubiquitin and initiates pathways in yeast cells that allow their growth on selective media or detection by color. An explicit advantage of this YTH system is its capability to uncover transient interactions, which might remain unnoticed with other techniques.

A large set of bait vectors encoding cDNA fragments of Ca_V_2 Ca^2+^ channel α1 subunits were constructed in our laboratory to identify, confirm, and characterize so far unknown Ca^2+^ channel interaction partners [44–46]. The selection of bait constructs followed our aim to identify specific Ca_V_2.2 interactors and to prove or to exclude the identified candidates as common Ca_V_2-family interactors. Since expression of full-length α_1_ subunit cDNAs as functional baits in yeast was not possible, we constructed bait vectors encoding single domains together with their adjacent intracellular regions loop regions. These constructs ensure that putative interaction partners might not only bind to regions of limited length, but may also require multiple sites.

As a control and to verify correct structure and integration of Ca_V_ Ca^2+^ channel α_1_ fragments into membranes, we expressed domain I and the loop I–II region of Ca_V_1.2 together with the ß_2_ subunit in yeast cells. This interaction has been well-characterized in the literature and was narrowed down to the AID and BID sites. Our control experiments led to a robust growth of yeast on selective media, indicating that the Ca_V_ constructs used for the screening experiments were folded correctly and maintained their capability to interact with other proteins in the YTH system.

Screenings were performed with mouse brain cDNA libraries and resulted in the identification of more than 30 candidates. All these putative interaction partners were checked for false-positive candidates by confirmation assays. Finally, our YTH screenings ended up by identifying six novel interaction partners, tetraspanin-13 (TSPAN-13), reticulon 1 (RTN1), member 1 of solute carrier family 38 (SLC38), prostaglandin D2 synthase (PTGDS), transmembrane protein 223 (TMEM223) and transmembrane BAX inhibitor motif 3 (TMBIM3) containing protein.

### Newly identified Ca_V_2.2 interaction partners

*Tetraspanin-13 (TSPAN-13)*. TSPAN-13 belongs to the tetraspanin family of membrane proteins consisting of four transmembrane segments and are characterized by a long extracellular loop (LEL) with a conserved CCG motif [47,48]. Tetraspanins are ubiquitously expressed and interact with a multitude of other membrane proteins thereby forming the so-called tetraspanin web. Those tetraspanin-enriched microdomains have been shown to affect intracellular trafficking, cell migration, fusion, and signaling in general.

We constructed a series of TSPAN-13 deletion mutants to narrow down the interaction site with domain IV of Ca_V_2.2. These studies showed that interaction still occurred even when the last two transmembrane segments, the LEL and the C-terminus were removed. C-terminal EGFP-tagged TSPAN-13 constructs confirmed membrane localization of all mutants. The deletion of the LEL motif is of particular importance since this region has been shown to be of critical importance for facilitating protein–protein interactions [49–51]. In contrast to these studies, the mutation of conserved amino acids within transmembrane segment S2 abolished the interaction with Ca_V_2.2. Because of the hydrophobicity of Ca_V_2.2 domain IV and S2 of TSPAN-13, we assume a hydrophobic interaction between transmembrane segments of both proteins.

It should be noted that the TSPAN-13-Ca_V_2.2 interaction has not been identified by proteomic approaches [42]. Hydrophobic interactions between transmembrane segments might be broken up when applying stringent solubilization or co-immunoprecipitation conditions. Thus, we are convinced that only the combination of different methodological approaches, each with their individual strengths, results in a more complete map of the Ca_V_2.2 interactome.

*Reticulon 1 (RTN1)*. Our screenings identified RTN1 as an interactor of domain IV of all three members of the Ca_V_2-family. RTN1 demonstrates a broad expression in membranes of intracellular compartments with a higher density around the nuclei but also in the plasma membrane [45,52]. RTN1 is the only interactor of our YTH screenings that was also identified by a proteomic MS approach [42]. The physiological functions of RTN1 are largely unknown, but there are several reports linking RTN1 with neuropathophysiological conditions like Alzheimer´s disease, cerebral ischemia and apoptosis [53–55]. It should be noted that the splice variant RTN1-C expressed in neuronal and neuroendocrine tissue has been shown to bind to SNARE proteins and form part of the Ca_V_2 channel nanodomain [56].

*Member 1 of solute carrier family 38 (SLC38)*. SLC38 solely interacts with domain IV of Ca_V_2.2, but not with other Ca_V_2 channels. It is predominantly expressed in GABAergic neurons and is involved in the uptake of glutamine, a precursor of GABA synthesis [57,58]. The transporters of the SLC38 family are typically regulated by diverse stimuli such as depletion of amino acids, by osmolarity or by hormones. Transport capacity is increased by trafficking to and fusion of SLC containing vesicles with the plasma membrane [59]. Since these processes depend on local elevation of Ca^2+^, a functional link with Ca^2+^ channel activity may be hypothesized.

*Prostaglandin D2 synthase (PTGDS)*. This protein interacted with domain IV of all Ca_V_2 channels, but not with their C-termini. Thus, PTGDS is an example for a Ca_V_2-family-specific interactor in contrast to other identified candidates. PTGDS is responsible for the final synthesis step from the intermediate prostaglandin H2 to active prostaglandins such as PGD2. Furthermore, it has been shown that PTGDS is involved in regulation of the peripheral nervous system myelination, where Ca_V_2.2 is highly expressed [60]. It was demonstrated that neuregulin 1 type III induces expression of PTGDS and activation of Gpr44 and thereby contributes to myelin formation.

*Transmembrane protein 223 (TMEM223)*. Also, TMEM223 interacted with domain IV of all Ca_V_2 channels. Unfortunately, there are no publications that give us insight on possible functions or links to Ca_V_ channels.

*Grina/TMBIM3*. This interactor is highlighted in a separate chapter below.

### Modulation of Ca_V_2.2 function by novel interaction partners

Modulation of the current through the Ca_V_2.2 channel complex is summarized in Table 2. Most straightforward downregulation of calcium entry results from suppression of current amplitude. Such effect is caused by channel interaction with PTGDS, TMEM223, and Grina/TMBIM3 [45,46]. The negative effect of TMEM223 is further amplified by an increase in the extent of cumulative inactivation by an action potential train. SLC38 suppressed Ca^2+^ entry during the Ca_V_2.2 channel opening by enhancing the cumulative channel inactivation during an action potential train. Possible importance of such regulation is underlined by the fact that such mode of channel activation mimics physiological conditions. TSPAN-13 accelerated the activation of both current activation and inactivation. These effects counteract each other, so the final result will depend on the actual mode of channel activation under physiological conditions. More detailed analysis [44] revealed that TSPAN-13 reduced the efficiency of the coupling between activation of the voltage sensor and pore opening, thereby supporting a negative modulatory effect of this protein. RTN1 did not alter any of analyzed electrophysiological parameters. Most apparent was a negative modulation of Ca^2+^ entry by Grina/TMBIM3 protein.

**Table 2.** Interaction of novel interaction partners with the Ca_V_2.2 channel tested by means of electrophysiology. Meaning of symbols: ND – not determined; np – not published (unpublished data of manuscript authors); ↔ non-significant effect; ↑ – statistically significant enhancement (acceleration of activation/inactivation kinetics, increase in extent of cumulative inactivation, increased current facilitation by prepulse); ↓ – statistically significant suppression (lower current amplitude, slowed kinetics of activation, smaller extent of cumulative inactivation, reduced current facilitation by prepulse). Modulations resulting in “loss-of-function” are highlighted in red, “gain-of-function” modulations are marked in green.

Protein	Current amplitude	Voltage dependence of activation/inactivation	Kinetics of current activation/inactivation	Cumulative inactivation	Prepulse facilitation
^a^TSPAN-13	**↔**	**↔**/ND	↑/**↑**	**↔**	**↔**
^b^RTN1	**↔**	**↔/↔**	**↔**/ND	**↔**	**↔** np
^b^SLC38	**↔**	**↔/↔**	**↔**/ND	**↑**	**↔** np
^b^PTGDS	**↓**	**↔/↔**	**↔**/ND	**↔**	**↔** np
^b^TMEM223	**↓**	**↔/↔**	**↔**/ND	**↑**	**↑** (p = 0.058; np)
^c^Grina/TMBIM3	**↓**	**→** np/ND	**↓** np/↔ np	**↓**	**↑**
^c^Gβγ	**↓**	**→** np/ND	**↔**/ND	**↓**	**↑**

a – [44], b – [45], c – [46]

**Figure 3.**
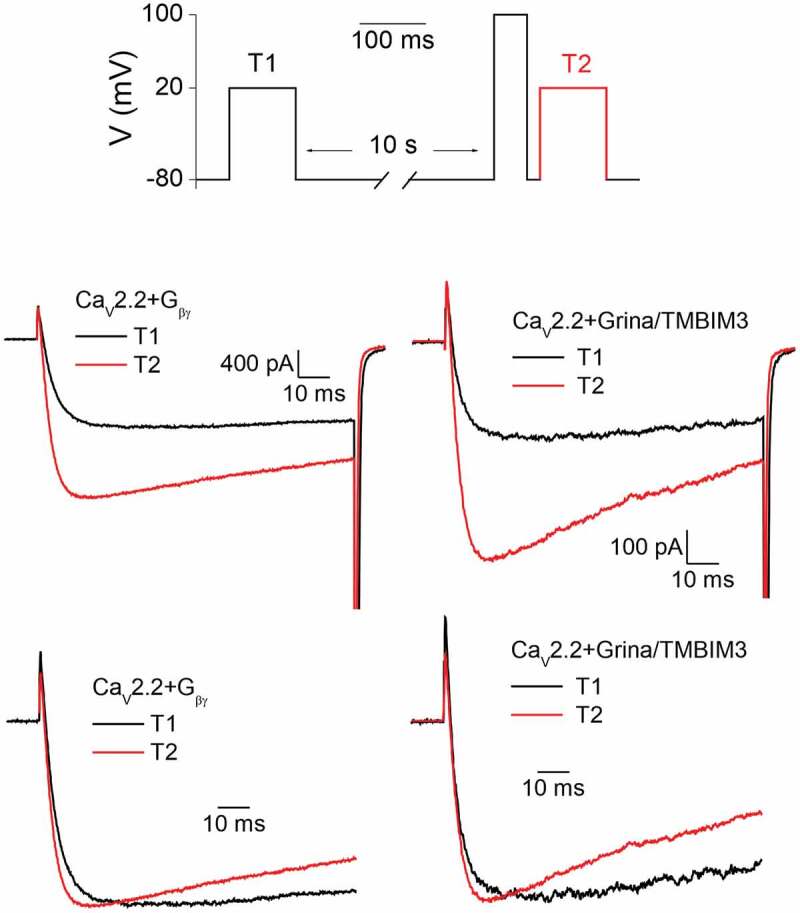
Prepulse facilitation protocol used in CHO cells stably transfected with human α1-Ca_V_2.2, α_2_δ-1, and β1 subunits coexpressed with Gβγ subunits of G proteins (left) or with Grina/TMBIM3 (right) relieved current inhibition and accelerated activation kinetics in a similar manner [46]. In the bottom row, current traces recorded, before (black) and after (red) depolarizing prepulse were normalized to the same amplitude to facilitate comparison of current kinetics.

Coexpression of the Grina/TMBIM3 protein together with the Ca_V_2.2 channel decreased the peak Ca^2+^ current density by approximately 54% [46] (see also Figure 3), i.e., to an extent comparable to those caused by coexpression of PTGDS (57%) and higher than that caused by coexpression of TMEM223 (67%) [45]. Negative modulatory effects of Grina/TMBIM3 on the Ca^2+^ current were further enhanced by a shift of the voltage dependence of current activation toward more positive membrane potentials and by slowing the kinetics of current activation [46] (see also Figure 3). In contrast to TMEM223, Grina/TMBIM3 mitigated the cumulative inactivation of the Ca^2+^ current, caused by an action potential train [45,46]. Alleviation of inhibition, caused by repetitive depolarization, goes in hand with a highly significant prepulse facilitation by Grina/TMBIM3, but not by TMEM223 (p = 0.0065 and p = 0.058, respectively, compared to facilitation in the absence of the respective interactor; unpaired Student’s t-test) [45]; and Table 2.

**Figure 4.**
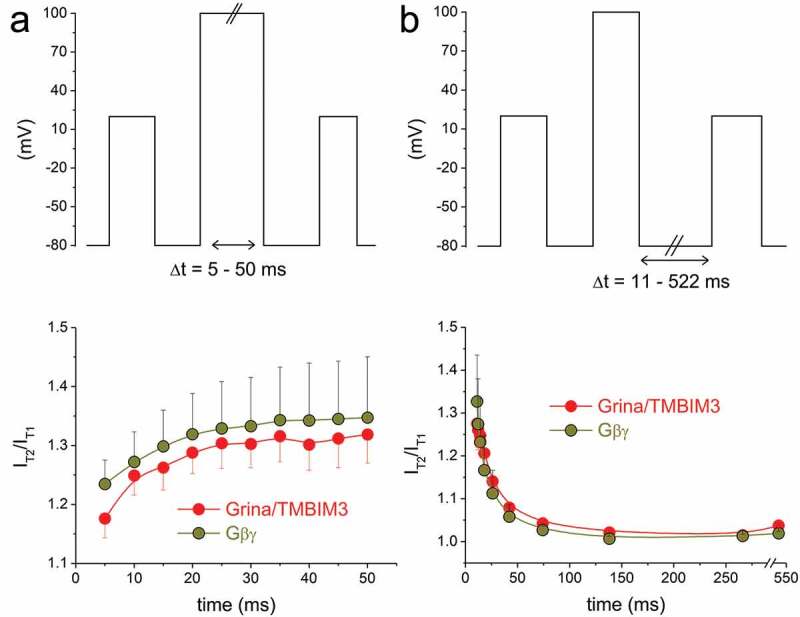
Prepulse facilitation was applied in CHO cells stably transfected with human α1-Ca_V_2.2, α_2_δ-1, and β1 subunits coexpressed with Gβγ subunits of G proteins (olive) and with Grina/TMBIM3 protein (red). In panel (a) length of depolarizing pulse was gradually increased and prepulse facilitation evolved with increasing pulse length due to unbinding of Gβγ subunits from the channel. In panel (b) time intervals after depolarizing pulses were increased and prepulse facilitation faded away due to rebinding of Gβγ subunits. In both paradigms kinetics of effects was similar.

Facilitation of Ca^2+^ currents by brief high depolarizations was reported for several voltage- dependent Ca^2+^ channel types, but most prominent enhancement of current amplitude was shown for the Ca_V_2.2 channel. The underlying mechanism was extensively investigated and attributed to unbinding of Gβγ subunits. As already mentioned, binding of Gβγ subunit to α1 subunit of the Ca_V_2.2 channel is accompanied by a decrease in current amplitude, rightward shift of current–voltage relation, slowed activation kinetics, suppressed cumulative inactivation, and pronounced prepulse facilitation ([30,61–63] and Table 1). These characteristics exactly matched those of current modulation by Grina/TMBIM3, reported by our group ([46] and Table 2). Results summarized in Table 2 were obtained from various experimental objects, including freshly isolated neurons (guinea pig cholinergic neurons [30]), neuronal cell model (NG108-15 cells [62]), recombinant expression systems (HEK293 cells [61] and *Xenopus laevis* oocytes [63]). Results reported for Grina/TMBIM3 were obtained from the recombinant expression model using CHO cells; therefore, similarity was verified by expression of recombinant Gβγ subunit in this system, too ([46] and Table 2). Further, for alleviation of Gβγ inhibition, optimal length of depolarizing prepulse is required. The time course of development of prepulse facilitation with increasing length of the prepulse reflects the kinetics of Gβγ unbinding from the Ca_V_2.2 α_1_ subunit. This kinetics was virtually identical when Grina/TMBIM3 or Gβγ were coexpressed together with the Ca_V_2.2 channel (Figure 4a). *Vice versa*, following depolarizing prepulse, channels recover from facilitation due to rebinding of Gβγ subunits to the channel’s α_1_ subunit when membrane potential was maintained at resting potential values. Time course of recovery from facilitation can be observed as a function of the length of repolarization period which follows conditioning prepulses (Figure 4b). Again, this kinetics was similar to whether Grina/TMBIM3 or Gβγ were coexpressed together with the Ca_V_2.2 channel. All these observations lead to the suggestion, that Grina/TMBIM3 and Gβγ subunit of G proteins may share a common mechanism of Ca_V_2.2 modulation [46]. The same conclusion cannot be made for TMEM223, as the only feature of current inhibition shared with its inhibition by G proteins is nearly a significant prepulse facilitation (Table 2). Such a moderate effect may suggest that TMEM223 facilitated channel inhibition by G proteins native to CHO cells used in experiments rather than a direct interaction of this protein with the channel.

To achieve closer insights into possible mechanism of channel modulation by Grina/TMBIM3, a structural analysis of this protein and its putative interaction site with Ca_V_2.2 α_1_ subunit is highly desirable.

### New insights on the structure of Grina/TMBIM3

Grina/TMBIM3 was initially characterized as an NMDA-receptor associated subunit, but increasing evidence from recent literature indicates that Grina/TMBIM3 is involved in the unfolded protein response and controls apoptosis *via* regulation of Ca^2+^ homeostasis [64,65]. Grina/TMBIM3 contains a conserved BAX inhibitor-1 motif that led to the renaming of Grina to TMBIM3 (transmembrane BAX inhibitor motif 3) and defines Grina/TMBIM3 as part of a small family of proteins comprising at least seven mammalian members with anti-apoptotic properties. Bioinformatics predicts a transmembrane protein with seven transmembrane segments and a large intracellular N-terminus. However, experimental data also suggest that at least some TMBIM members have a six transmembrane topology placing the N- and C-termini to the cytosol [66]. This discrepancy prompted us to map the topology of Grina/TMBIM3, in particular to determine the disputed localization of the C-terminus.

**Figure 5.**
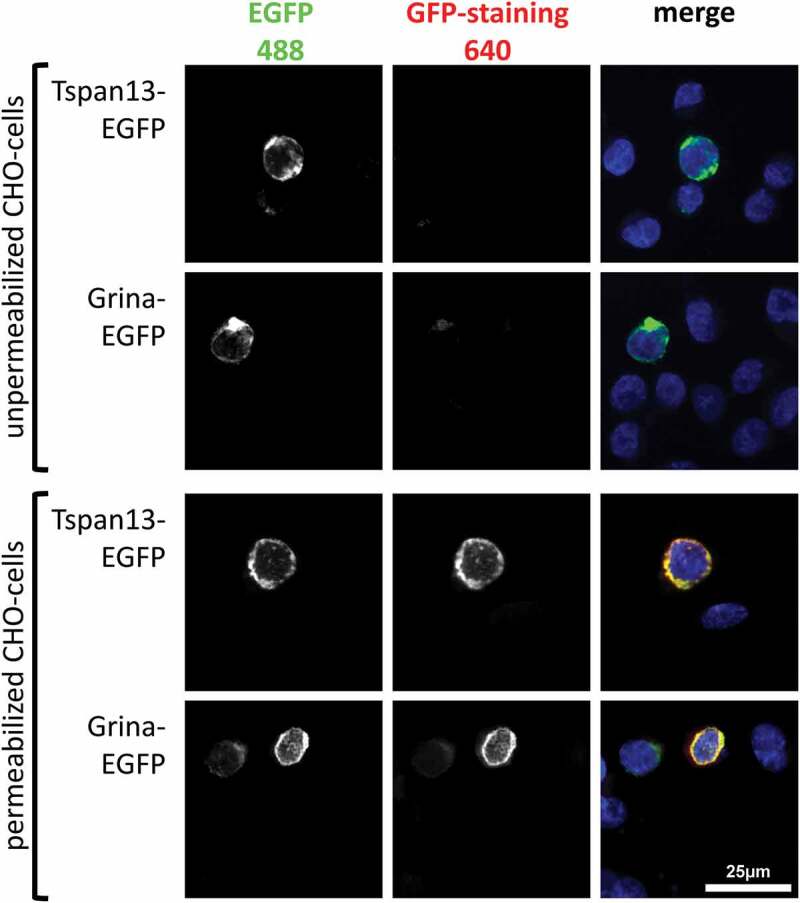
CHO cells were transfected with C-terminal EGFP-tagged Grina/TMBIM3 and as a control with EGFP-tagged Tspan13. Tspan13 is a four transmembrane protein with cytosolic oriented N- and C-termini. Following transfection, both proteins show the same staining pattern, demonstrating that Grina/TMBIM3 is localized in the plasma membrane. However, only permeabilized cells allow an antibody staining of EGFP-tag indicating a cytosolic orientation of the Grina/TMBIM3 C-terminus.

To achieve closer insights into the membrane topology of Grina/TMBIM3, we fused EGFP in frame to the C-terminus of Grina/TMBIM3 and transfected the corresponding plasmid in CHO cells. As a control, we used a C-terminal EGFP-tagged tetraspanin-13 (Tspan13), which shows a clearly visible plasma membrane localization with cytosolic N- and C-termini [44]. Twenty-four hours post-transfection both EGFP-tagged proteins were visible at the plasma membrane (Figure 5). To determine the localization of the C-termini either as extra- or intracellular, the transfected CHO cells were also stained with an antibody against EGFP. In this experiment, only cells, permeabilized with 0.3% TritonX100 Grina/TMBIM3 and Tspan13, could be stained at the plasma membrane with the EGFP-antibodies (Figure 5). This study clearly shows that the Grina/TMBIM3 C-terminus is not accessible to the antibody from the outside and only permeabilization allows antibody staining. These data are consistent with that from Carrara and colleagues and argue for a cytosolic orientation of both, N- and C-termini of TMBIM proteins, and possibly for a six transmembrane topology [66].

### Putative mechanism of Ca_V_2.2 channel modulation by Grina/TMBIM3 protein

Whole cell currents through Ca_V_2.2 channels in the presence of coexpressed Grina/TMBIM3 has characteristics of a “reluctant” gating state: current amplitude is suppressed, channel activation requires higher depolarization and its kinetics is slowed, cumulative inactivation caused by an action potential train is relieved, and depolarizing prepulses shift gating characteristics into those typical for the “willing” state [46]. Further, kinetics of facilitation onset and recovery from facilitation are similar to those evoked by Gβγ subunits. The effects of Gβγ subunits and Grina/TMBIM3 are not additive [46], suggesting shared binding sites.

Grina/TMBIM3 was shown to directly interact with domain IV of the channel protein [46], however, this study did not exclude the existence of additional interaction sites. Considering a low efficiency of G protein-dependent channel inhibition mediated by the C-terminus, it is likely that such sites may exist. As Grina/TMBIM3 is a membrane-localized protein with six-transmembrane segments and intracellular N- and C-termini, when colocalized in a cell membrane with Ca_V_2.2, it may interact with outer segments and/or intracellular loops of the Ca_V_2.2 α_1_ subunit. However, it is unlikely that Grina/TMBIM3 could compete with intracellular Gβγ subunits for binding in putative GPBP [40].

The David Yue group suggested that Gβγ subunits alter intrinsic gating properties of the channel possibly by interacting with its voltage sensor [67]. This notion was further supported by a study showing that a G177E mutation in the IS3 segment of Ca_V_2.2, which may stabilize the closed state by introducing a negative charge interacting with positive charges in the IS4 segment, transfers the channel into the “reluctant” gating state [68]. It is not clear whether membrane localization of Grina/TMBIM3 would allow an interaction with the voltage sensor, nevertheless, a detailed analysis of gaiting currents in the presence and absence of the protein may offer further insight into the putative mechanism of Grina/TMBIM3 action.

## Conclusions

Ca_V_2.2 channels represent a prominent target for pharmacological treatment of chronic and neuropathic pain. Multiple pathways may be causative for a negative modulation of Ca^2+^ entry: inhibition of the channel, inhibition of channel targeting into the plasma membrane or disruption of the signaling complex, which activates exocytosis (Figure 1). Newly identified interaction partners, presented in this review, directly target the channel complex itself. Five out of six proteins negatively modulate the current through Ca_V_2.2 (Table 2). Three of them, PTGDS, TMEM223, and Grina/TMBIM3, suppressed the amplitude of the current activated by a rectangular depolarizing pulse by about 50% [45,46]. Two proteins, TMEM223 and Grina/TMBIM3, modulate the Ca^2+^ current in a more complex way, suggesting a state-dependent interaction with the channel. TMEM223 may preferably interact with the inactivated channel state, as it enhanced the cumulative voltage-dependent inactivation of the channel and there is a strong tendency to relieve the inhibition by pushing all channels into an open state [45]. In contrast, inhibition of the current by Grina/TMBIM3 has all characteristics of an interaction with the closed channel [46]: slowed activation kinetics, shift of voltage-dependence of channel activation toward more depolarized potentials, relieve of current inhibition by transfer of all channels into an open state and a reduction of the cumulative voltage-dependent inhibition of the channel. Such modulation is a signature of channel inhibition by G-proteins activated by agonists of opioid receptors. The fact, that Grina/TMBIM3 shares a common mechanism of Ca_V_2.2 channels inhibition with agonists of opioid receptors like morphine, suggests that alternative channel inhibitors mimicking opioids may be designed for pain therapy.
